# Intragenic DNA methylation of *PITX1* and the adjacent long non-coding RNA *C5orf66-AS1* are prognostic biomarkers in patients with head and neck squamous cell carcinomas

**DOI:** 10.1371/journal.pone.0192742

**Published:** 2018-02-09

**Authors:** Verena Sailer, Arthur Charpentier, Joern Dietrich, Timo J. Vogt, Alina Franzen, Friedrich Bootz, Dimo Dietrich, Andreas Schroeck

**Affiliations:** 1 Department of Pathology and Laboratory Medicine, Weill Cornell Medicine, New York, NY, United States of America; 2 Caryl and Israel Englander Institute for Precision Medicine, Weill Cornell Medicine, New York, NY, United States of America; 3 University Hospital Bonn, Department of Otolaryngology, Head and Neck Surgery, Bonn, Germany; University of Cincinnati College of Medicine, UNITED STATES

## Abstract

**Background:**

Patients with squamous cell cancer of the head and neck region (HNSCC) are at risk for disease recurrence and metastases, even after initial successful therapy. A tissue-based biomarker could be beneficial to guide treatment as well as post-treatment surveillance. Gene methylation status has been recently identified as powerful prognostic biomarker in HNSCC. We therefore evaluated the methylation status of the homeobox gene *PITX1* and the adjacent long intergenic non-coding RNA (lincRNA) *C5orf66-AS1* in publicly available datasets.

**Methods:**

Gene methylation and expression data from 528 patients with HNSCC included in The Cancer Genome Atlas (TCGA, there obtained by using the Infinium HumanMethylation450 BeadChip Kit) were evaluated and methylation and expression levels of *PITX1* and lincRNA *C5orf66-AS1* was correlated with overall survival and other parameters. Thus, ten beads targeting *PITX1* exon 3 and three beads targeting lincRNA *C5orf66-AS1* were identified as significant candidates. The mean methylation of these beads was used for further correlation and the median was employed for dichotomization.

**Results:**

Both *PITX1* exon 3 and lincRNA *C5orf66-AS1* were significantly higher methylated in tumor tissue than in normal adjacent tissue (NAT) (*PITX1* exon 3: tumor tissue 58.1%, NAT: 31.7%, p<0.001; lincRNA *C5orf66-AS1*: tumor tissue: 27.4%, NAT: 18.9%, p<0.001). In a univariate analysis, hypermethylation of both loci was significantly associated with the risk of death (univariate: exon 3: Hazard ratio (HR): 4.97 [1.78–16.71], p = 0.010, lincRNA *C5orf66-AS1*: Hazard ratio (HR): 12.23 [3.01–49.74], p<0.001). *PITX1* exon 3 and lincRNA *C5orf66-AS1* methylation was also significantly correlated with tumor localization, T category, human papilloma virus (HPV)-negative and p16-negative tumors and tumor grade. Kaplan-Meier analysis showed, that lincRNA *C5orf66-AS1* hypomethylation was significantly associated with overall survival (p = 0.001) in the entire cohort as well in a subgroup of HPV-negative tumors (p = 0.003) and in patients with laryngeal tumors (p = 0.022).

**Conclusion:**

Methylation status of *PITX1* and even more so of lincRNA *C5orf66-AS1* is a promising prognostic biomarker in HNSCC, in particular for HPV-negative patients. Further prospective evaluation is warranted.

## Introduction

Squamous cell carcinoma of the head and neck region (HNSCC) is a common malignancy with 5-year survival rates ranging from 61% for laryngeal cancers to 63% for cancers of the oral cavity and pharynx [1]. For 2017, 63,030 new cases are estimated in the United States and 13,360 patients are estimated to die of cancer-related causes [2].

Besides cigarette smoking and alcohol, infection with high-risk human papilloma viruses (HPV) like HPV16 has now been well established as an important risk factor in HNSCC development. HPV-positive tumors are predominantly located in the oropharynx and the incident of these tumors is increasing [3]. Moreover, since a positive HPV-status is associated with better overall survival, HPV-positive tumors have now been recognized as a distinct entity in the new 8^th^ edition of the TNM-classification [4, 5]. Molecular features of an additional “atypical” HNSCC subtype, i.e. female patients with oral cavity tumors, who are HPV-negative and have no smoking history, have recently been described. These tumors exhibit a CpG island methylator phenotype (CIMP) with striking genomic stability, but are characterized by gene silencing through methylation events [6].

Since recurrence and metastasis is common after initial treatment, biomarkers need to be identified that can aid in stratification of treatment and surveillance after first-line curative therapy, in particular in HPV-negative patients [7]. More systemic therapeutic regimens than ever are now available for HNSCC patients, including immunotherapy [8]. Therefore, a prognostic biomarker can have a real impact in terms of influencing therapeutic strategy. A comprehensive review on molecular biomarkers in head and neck cancer is provided by Juodzbalys *et al*. [9].

Aberrant DNA methylation is found frequently in cancer and the methylation status of various functional genomic loci, e.g. promoters and bodies (exons and introns) of translated genes or genes encoding long non-coding RNAs (lncRNAs), can be used as biomarker. LncRNAs are longer than 200 base pairs and do not code for proteins but can influence cancer development and progression [10]. They can be classified as, amongst others, sense/antisense or long intergenic. Long intergenic non-coding RNA are localized between two genes and are involved in chromatin remodeling [11]. Sense/antisense non-coding RNA overlap one or more exons of other transcripts on the same (sense) or opposite (antisense) strand [12]. In addition, DNA methylation of long non-coding RNA promoter regions occurs during cancer development [13]. Promoter methylation of paired like homeodomain 2 (*PITX2*) transcription factor is a well-established and validated prognostic biomarker in various malignancies, including carcinomas of the prostate, breast, biliary tract, and lung [14–20]. Recent publications suggest a role of methylation status of *PITX3* and *PITX2* and adjacent long non-coding RNA (*PANCR*) as promising new biomarkers in HNSCC [21, 22]. Another gene in this family, the transcription factor *PITX1*, might exhibit tumor-suppressing properties by regulating p53 transcription [23]. Moreover, *PITX1*-mediated p120RasGAP activation negatively influences Ras signaling [24, 25]. These data point to a role of *PITX1* as potential tumor suppressor and dysregulation could influence cancer development. A germline single nucleotide polymorphism in the *PITX1* gene has been identified as a susceptibility locus for colorectal cancer [26]. Like other genes in this family, *PITX1* is an essential transcription factor in embryogenesis and is involved in mouth and hindlimb formation and pituitary development [27–29]. Chromosomal rearrangements involving the *PITX1* gene result in Liebenberg syndrome with partial arm-to-leg transformation [30]. *PITX1* is located on chromosome 5q31, in very close proximity to the long intergenic non-coding RNA *C5orf66-AS1*, which is also called *Epist* [31, 32]. So far, only few studies have investigated lincRNA C5orf66-AS1. Feng *et al*. found a significant difference of expression when comparing tissue of oral squamous cell cancer with normal mucosa [32]. Yu et al. also observed decreasing expression of lincRNA C5orf66-AS1 from normal pituitary tissue to non-invasive to invasive pituitary null cell adenomas. They performed an additional in silico analysis and predicted *PITX1* as target gene of lincRNA C5orf66-AS1. Interestingly, *PITX1* expression is also decreased in several malignant tumors like gastric, colon and bladder cancer as well as HNSCC [25, 33, 34]. Reduced *PITX1* expression is associated with treatment response in the latter [34]. The molecular mechanisms of the reduced expression of lincRNA C5orf66-AS1 and PITX1 in tumor tissue are incompletely understood and might be the result of epigenetic regulation. Aberrant PITX1 methylation has been found in salivary gland adenoid cystic carcinoma and is associated with survival in clear cell renal cell carcinomas [35, 36].

In the present study, methylation status of both *PITX1* and lincRNA *C5orf66-AS1* as well as corresponding mRNA levels were investigated and correlated with overall survival and clinicopathological parameters to evaluate its potential as a prognostic biomarker in HNSCC.

## Methods

### Ethical approval

The present study is based entirely upon data generated by the TCGA research network (www.cancergenome.nih.gov). All patients included in TCGA have been enrolled following strict human subjects protection guidelines, informed consent and IRB (Institutional Review Board) review of protocols. All patients provided informed consent (written).

### Patients

527 head and neck squamous cell carcinoma patients included in the TCGA HNSC cohort with follow-up data were included and analyzed retrospectively. Data from normal adjacent tissues (NATs) were available for 50 of these patients. Data for *PITX1* mRNA was available for 540 patients. Methylation data for both gene loci was available for 578 samples each. Clinicopathologic parameters of the whole cohort are listed in S1 Table. Survival was defined as time to death by any cause (overall survival, OS) and censored after five years (60 months), in order to exclude deaths that were not HNSCC-related [6].

### DNA Methylation, mRNA and lncRNA expression, and HPV/p16 analyses

**Fig 1:**
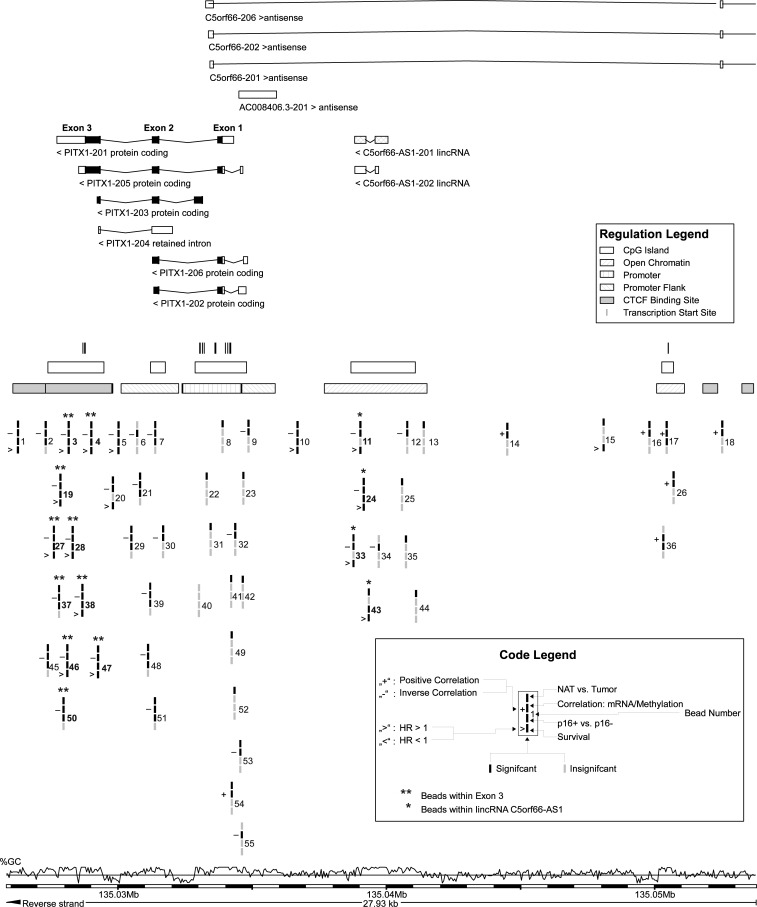
Organization of the *PITX1* and *C5orf66-AS1* genes. Genomic organization of the *PITX1* gene and *C5orf66-AS1* genes and target regions of Illumina HumanMethylation450 BeadChip beads. Significant beads were grouped within the homeobox containing exon 3 and lincRNA *C5orf66-AS1*. Grouped beads are labeled with ‘*’ and ‘**’, respectively. The information was taken from Ensembl Homo sapiens version GRCh38.p10.

Data was processed as described previously [21]. In brief, gene methylation data were downloaded from the UCSC Xena browser (www.xena.ucsc.edu). TCGA methylation analysis has been performed using the Infinium HumanMethylation450 BeadChip (Illumina, Inc., San Diego, CA, USA). Methylation levels (Beta-values) were calculated as previously described [37–39]. In brief, Beta-values were defined as: Beta = (Intensity_Methylated) / (Intensity_Methylated—Intensity_Unmethylated + α) [40]. The constant offset α was set to 0. Beta-values (values between 0 and 1) were multiplied with the factor 100% in order to shown percent methylation (0 to 100%). The following Illumina beads (numbered according to Fig 1) were investigated: cg00874891 (1), cg15922699 (2), cg04223420 (3), cg12673103 (4), cg18606375 (5), cg21614303 (6), cg08373003 (7), cg01966335 (8), cg24495017 (9), cg09713684(10), cg18443359 (11), cg14994060 (12), cg16258223 (13), cg11591267 (14), cg17034591 (15), cg04847174 (16), cg15528437 (17), cg03827835 (18), cg02037307 (19), cg05171952 (20), cg02213684 (21), cg02948884 (22), cg25648267 (23), cg15105206 (24), cg09839170 (25), cg01213519 (26), cg22488797 (27), cg07274716 (28), cg03347590 (29), cg23064601 (30), cg00089224 (31), cg19802165 (32), cg01830023 (33), cg24462476 (34), cg13441766 (35), cg15421305 (36), cg26509691 (37), cg00396667 (38), cg13715631 (39), cg11512280 (40), cg23341163 (41), cg11788465 (42), cg03654472 (43), cg06566775 (44), cg12622597 (45), cg22827250 (46), cg04101060 (47), cg02495310 (48), cg26972058 (49), cg08206318 (50), cg02100373 (51), cg08255782 (52), cg25330797 (53), cg12129103 (54), cg06933574 (55).

In addition, RNAseq data was obtained. RNAseq data generation has been described before for TCGA [41]. C5orf66-AS1 (ENSG00000249082.1) lincRNA expression levels were obtained from TANRIC (http://ibl.mdanderson.org/tanric/_design/basic/download.html) [42]. Prevalence of HPV-infection was reported by using two different methods: p16 status by immunohistochemistry (termed p16-positive/negative) or viral integration assessed by RNAseq data (termed HPV-positive/negative).

### Statistics

SPSS version 24 (SPSS Inc., Chicago, IL, USA) was used for statistical analysis. Kaplan-Meier and Cox proportional hazard regression analyses were performed for survival analyses. Correlation between different parameters was tested using the Spearman’s rank correlation coefficient (ϱ). Mann-Whitney-*U* test, one-way analysis of variance (one-way ANOVA), and Fisher’s exact test were used for comparison between groups. P-values lower than 0.05 were considered significant.

## Results

### Identifying significantly methylated genomic regions

Fifty-five bead types for both gene loci were investigated (Fig 1) in this cohort of n = 527 patients. For *PITX1*, ten bead types were identified, which provided clinically significant information and matched to CpG islands on exon 3. Four beads were identified for lincRNA *C5orf66-AS1*. Two beads (cg18443359, cg15105206) are located within the second exon and one bead (cg03654472) targets the intron of *C5orf66-AS1-201*. One bead (cg01830023) is located 32 base pairs downstream from the antisense RNA.

Fifty out of 55 beads were differentially methylated between normal and tumor tissue. Inverse correlation between mRNA expression and methylation was found in 32/55 beads. Twenty-five out of 55 beads were significantly differential expression between HPV-positive and HV-negative cases. When evaluating methylation as continuous variable, 17/55 beads showed significant association with overall survival.

Of the ten beads selected for analysis in *PITX1* exon 3, all but two beads were significantly associated with overall survival. All three beads selected for analysis in lincRNA *C5orf66-AS1* were significantly associated with overall survival. The mean methylation of the respective beads was used for subsequent analyses.

### *PITX1* exon 3 and lincRNA *C5orf66-AS1* methylation in tumor and normal adjacent tissue

**Fig 2:**
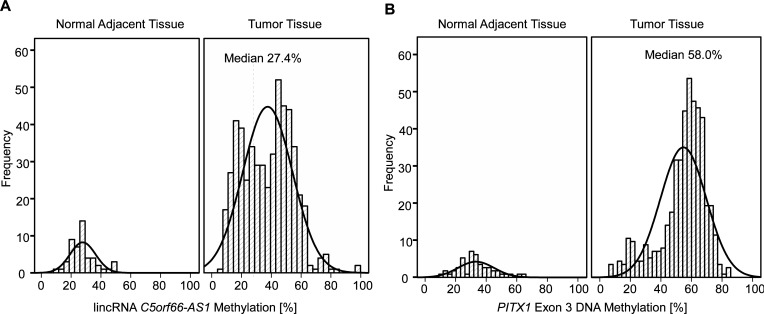
*PITX2* and *C5orf66-AS1* methylation distribution in tissues. Histogram showing the DNA methylation level (A: lincRNA C5orf66-AS1, B: *PITX1* exon 3) in normal adjacent tissue (n = 50) and HNSCC samples (n = 528). Tumor tissue DNA was methylated significantly higher compared to corresponding normal adjacent tissue (*PITX1* exon 3: p<0.001, lincRNA *C5orf66-AS1*: p<0.001; Mann-Whitney *U* test). In addition, two distinct peaks can be recognized in the DNA methylation level of lincRNA *C5orf66-AS1*, which are separated by the median cut-off.

Both *PITX1* exon 3 and lincRNA *C5orf66-AS1* were significantly higher methylated in tumor tissue than in normal adjacent tissue (*PITX1* exon 3: tumor tissue 58.1%, NAT: 31.7%, p<0.001; lincRNA *C5orf66-AS1*: tumor tissue: 27.4%, NAT: 18.9%, p<0.001; Fig 2). Fig 1 shows, that the methylation was investigated not at a promoter region, but at an intragenic region within *PITX1* exon 3. While methylation in promoter regions result in general in gene silencing, the functional consequence of intragenic methylation is less clearly defined. Both gene silencing and gene expression might ensue [43]. However, in the present dataset *PITX1* mRNA was also evaluated and was negatively correlated with *PITX1* exon 3 (Spearman’s ϱ = -0.347, p<0.001) as well as lincRNA *C5orf66-AS1* methylation (Spearman’s ϱ = -0.350, p<0.001). This indicates gene silencing as result of DNA methylation.

### Association of *PITX1* and lincRNA *C5orf66-AS1* methylation status with clinicopathological parameters

Methylation and expression of both *PITX1* mRNA and lincRNA *C5orf66-AS1* were significantly associated with tumor location, p16 expression, HPV-status and complete resection (R0) (S1 Table). HPV-status as assessed by RNAseq was available for 279 patients, 243 of whom were HPV-negative and 36 patients were HPV-positive. Expression of p16, which is regarded as surrogate marker for HPV infection [44], was available for 115 patients: 74 patients were p16-negative and 41 patients were p16-positive. In general, a higher methylation and a lower mRNA expression of genes was seen in tumors located in the larynx and oral cavity as well as in HPV-negative tumors.

### Association with overall survival

Cox proportional hazard analyses was performed using logarithmic value (base 2) of the methylation as continuous variable. The analysis as continuous variable without the introduction of a cut-off for result dichotomization allows for an accurate assessment of the biomarker performance without the risk of statistical artifacts resulting from overfitted cut-offs. In univariate analysis, both *PITX1* exon 3 and lincRNA *C5orf66-AS1* methylation was associated with an increased risk of death (hazard ratio (HR)_*PITX1*exon3_ = 1.63, 95%CI [1.12–2.38], p = 0.011; HR_lncRNA*C5orf66-AS1*_ = 1.47, 95%CI [1.16–1.86], p = 0.002). T category, nodal status and age were also associated with a risk of death in univariate analyses (p = 0.001, p = 0.016, p = 0.018, respectively; Table 1).

**Table 1:** Univariate and multivariate Cox proportional hazards analyses. Univariate and multivariate Cox proportional hazards analyses on overall survival in 528 HNSCC patients stratified according to DNA methylation levels and clinico-pathologic variables. Methylation of lincRNA *C5orf66-AS1* and *PITX1* exon 3 as well as expression of PITX1 mRNA and lincRNA C5orf66-AS1 were analyzed as continuous variates logarithmized to base 2.

	Univariate Cox		Multivariate Cox	
Variable	HR [95% CI]	*P*-value	HR [95% CI]	*P*-value
lincRNA C5orf66-AS1 methylation	1.47 [1.16–1.86]	0.002	1.22 [0.84–1.79]	0.29
*PITX1* exon 3 methylation	1.63 [1.12–2.38]	0.011	0.98 [0.55–1.77]	0.95
PITX1 mRNA expression	0.88 [0.76–1.01]	0.062	NA	NA
lincRNA C5orf66-AS1 expression	0.91 [0.85–0.98]	0.009	0.98 [0.88–1.08]	0.67
pT1/2 vs. pT3/4	0.54 [0.37–0.78]	0.001	0.42 [0.25–0.70]	0.001
pN0 vs. pN1/2	1.62 [1.09–2.40]	0.016	1.36 [0.87–2.13]	0.18
Age (continuous variable)	1.02 [1.00–1.03]	0.018	1.02 [1.00–1.04]	0.080
HPV (positive vs. negative)	0.36 [0.18–0.75]	0.006	NA	NA
p16 (positive vs. negative)	0.67 [0.18–2.48]	0.54	NA	NA
Grade (G1/2 vs. G3/4)	0.82 [0.56–1.18]	0.28	NA	NA
Surgical margin (positive vs. negative)	1.03 [0.81–1.31]	0.83	NA	NA
Localization (reference: oral cavity)		0.65		NA
Oropharynx	0.58 [0.29–1.15]	0.12	NA	NA
Hypopharynx	1.06 [0.15–7.65]	0.95	NA	NA
Larynx	0.98 [0.62–1.54]	0.97	NA	NA

NA: Not applicable; variate not included into multivariate analysis due to lack of significance in univariate analysis or largely missing data (HPV/p16)

Parameters that tested significant in univariate analyses were further analyzed in multivariate analyses. Here, T category remained as the only significant independent prognostic marker in multivariate Cox proportional hazard analysis.

**Fig 3:**
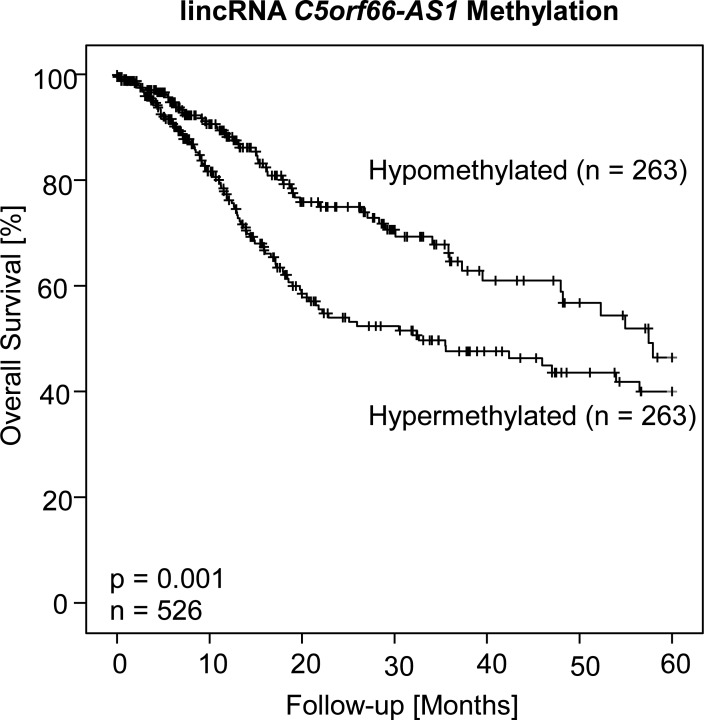
Kaplan-Meier survival analysis. Kaplan-Meier survival analysis of overall survival of 526 HNSCC patients stratified according to DNA methylation status of lincRNA *C5orf66-AS1*. Patient samples were classified as hypo- and hypermethylated applying the median methylation level (27.4%) for dichotomization of the methylation levels.

Methylation status was dichotomized in hypo- and hypermethylation using the median as cut-off for both gene loci in the entire cohort (median *PITX1* exon 3 methylation: 58.0%, median *C5orf66-AS1* methylation: 27.4%). The median was also used to dichotomize RNA levels (median PITX1 mRNA: 2585 [normalized counts], median C5orf66-AS1 lincRNA: 0.61). lincRNA C5orf66-AS1 hypomethylation was significantly associated with an improved overall survival (p<0.001, Fig 3). Kaplan-Meier curves showed a trend towards better survival in patients with high *PITX1* mRNA levels and hypomethylated *PITX1* exon 3 gene locus, but this result failed to be statistically significant (p = 0.21 and p = 0.12, respectively). In addition, lincRNA C5orf66-AS1 RNA levels were not associated with survival (p = 0.22). An optimized cut-off might have resulted in a significant result for all parameters, but was not applied in order to avoid overfitting.

**Fig 4:**
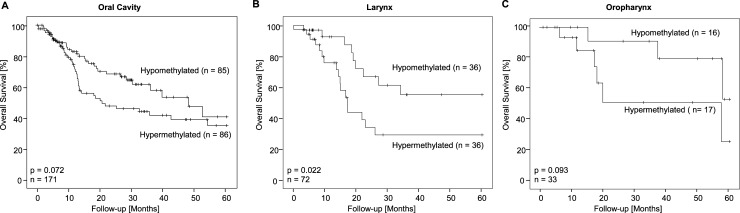
Kaplan-Meier survival analyses with regard to tumor localization. Kaplan-Meier survival analysis of overall survival in the subgroups of oropharyngeal (n = 33), oral (n = 171) and laryngeal (n = 72) carcinomas, respectively, included in the 528 HNSCC patients from the TCGA. Patients were stratified according to DNA methylation status of lincRNA *C5orf66-AS1*. Patient samples were classified as hypo- and hypermethylated applying the median methylation level for dichotomization of the methylation levels.

A subgroup analysis in different tumor locations (oral cavity, larynx and oropharynx) revealed a survival benefit for patients, whose laryngeal tumors were hypomethylated at the lincRNA *C5orf66-AS1* locus (p = 0.022, Fig 4). A trend towards significance in survival was seen in oral cavity and oropharynx tumors. In contrast to oropharynx tumors, both oral cavity and larynx tumors are in general not associated with HPV-infection [45]. Further sub-stratification of tumor location by HPV-status might have elucidated upon the survival benefit for these patients, but was prevented by the lack of HPV data.

**Fig 5:**
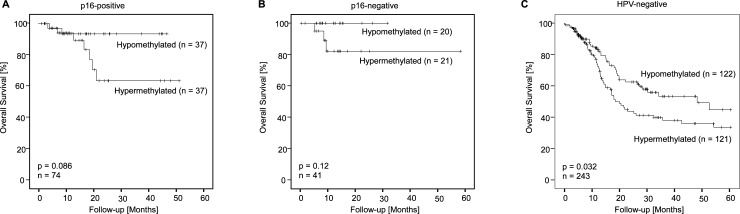
Kaplan-Meier survival analyses with regard to HPV-status. Kaplan-Meier survival analysis of overall survival in p16-positive (A), p16-negative (B), and HPV-negative (C) patients included in the 528 HNSCC patients from the TCGA. Patients were stratified according to DNA methylation status of lincRNA *C5orf66-AS1*. Patient samples were classified as hypo- and hypermethylated applying the median methylation level for dichotomization of the methylation levels.

When correlating lincRNA *C5orf66-AS1* hypo- and hypermethylation with p16 expression, a trend towards significance was seen in survival (p16-positive: n = 41, p = 0.086; p16-negative: n = 74, p = 0.12, Fig 5), which was supported by Kaplan-Meier curves, but ultimately failed to reach significance. This may be explained by the fact, that p16 expression was only available for 115 patients. However, additional stratification by HPV-status revealed a significantly better overall survival for patients with HPV-negative tumors and lincRNA *C5orf66-AS1* hypomethylation (n = 243, p = 0.032, Fig 5).

## Discussion

The present study evaluates for the first time the clinical utility of *PITX1* and lincRNA *C5orf66-AS1* methylation status as prognostic biomarker in HNSCC. Both parameters added significant information about risk of death in univariate analysis and hyomethylation of lincRNA *C5orf66-AS1* is associated with better survival, in particular in patients with negative HPV-status. While overall survival rates have improved in HNSCC, no improvement has been achieved for patients with laryngeal carcinoma, which is not thought to be associated with HPV infection [45–47]. As yet, only few prognostic biomarkers have been investigated in this patient cohort. For example, *in silico* analysis of expression data deposited in the Gene Expression Omnibus (GEO) identified spectrin, a cytoskleton protein, as a promising new biomarker [48]. Expression of the tyrosine kinase c-MET has been shown to be prognostic in oral squamous cell cancer, which is in general not associated with HPV-infection [49]. Expression of sonic hedgehog pathway related genes Gli-1 and Gli-2 was found to be associated with overall survival in a prospective study in patients with HPV-negative HNSCC [50]. Methylation status of lincRNA *C5orf66-AS1* could emerge as a useful prognostic biomarker to identify HPV-negative patients at risk for disease recurrence and metastases, who might benefit from additional therapy like immune checkpoint inhibitors.

The rationale for investigating *PITX1* and lincRNA *C5orf66-AS1* is strong, since both are lost in some tumor tissues. Moreover, genome-wide association studies have identified single nucleotide polymorphism associated with susceptibility of colorectal cancer in East Asian patients, notably rs647161 (A/C) on 5q31.1, which matches the position of *PITX1* [26]. This confirms a more prominent role of *PITX1* dysregulation in cancer development than previously thought. The present study indicates, that lincRNA C5orf66-AS1 might also be involved in tumorigenesis, likely by acting as (post)-transcriptional regulator of *PITX1*. LincRNA C5orf66-AS1 is down-regulated in esophageal squamous cell cancer and re-expression inhibits migration and invasion in vitro [51]. Further functional validation studies are needed to address this hypothesis. Loss of *PITX1* mRNA in the TCGA HNSCC dataset cannot be explained by genomic events like chromosomal perturbances or loss-of-function mutation. Only two out of 279 patients in the TCGA HNSCC dataset with HPV-status determined by RNAseq had a genomic alteration, one deletion and one -likely passenger- missense mutation, (cBioPortal, [52]). However, while this data is intriguing, a major limitation of our study is that the available RNAseq did not allow us to discriminate between different transcript variants. This would require a more detailed computational analysis and needs to be done in further studies [53]. Since our study is solely based on data generated by the TCGA network and we don’t have access to the tissues, we were not able to perform analyses of the specific transcripts. A detailed isoform-specific analysis should be performed in future studies.

*PITX1* is an upstream inducer of *RASAL1* and thus an important mediator of the Ras signaling pathway[54]. Loss of PITX1 and lincRNA C5orf66-AS1 by aberrant epigenetic regulation might point to an overactive Ras signaling pathway, thus identifying patients who could benefit from therapy targeting this pathway, e.g. sorafenib. In a recent analysis, a strong Ras signaling pathway was associated with short response to platinum-based chemotherapy plus cetuximab in HNSCC patients. The authors further investigated this finding in vitro and found indeed an overactive Ras signaling in a cetuximab-sensitive cell line [55]. The predictive potential of *PITX1* and lincRNA *C5orf66-AS1* methylation status should be investigated ideally in a prospective randomized clinical study.

This is the first study that investigates methylation status of *PITX1* and lincRNA *C5orf66-AS1* in patients with HNSCC. lincRNA *C5orf66-AS1* methylation is emerging as promising new prognostic biomarker to guide clinical treatment.

## Supporting information

S1 Table Associations of *C5orf66-AS1* and *PITX1* mRNA expression and methylation with clinicopathologic parameters. (XLSX)
